# Soil water management practices (terraces) helped to mitigate the 2015 drought in Ethiopia

**DOI:** 10.1016/j.agwat.2018.02.025

**Published:** 2018-05-31

**Authors:** Frédéric Kosmowski

**Affiliations:** CGIAR Standing Panel on Impact Assessment, ILRI, ​​P.O. Box 5689, Gurd Sholla Area, Addis Ababa, Ethiopia

**Keywords:** Soil water management practices, Climate-smart agriculture, Terraces, Contour bunds, Drought, Propensity score matching, Ethiopia

## Abstract

While the benefits of soil water management practices relative to soil erosion have been extensively documented, evidence regarding their effect on yields is inconclusive. Following a strong El-Niño, some regions of Ethiopia experienced major droughts during the 2015/16 agricultural season. Using the propensity scores method on a nationally representative survey in Ethiopia, this study investigates the effect of two widely adopted soil water management practices – terraces and contour bunds – on yields and assesses their potential to mitigate the effects of climate change. It is shown that at the national level, terraced plots have slightly lower yields than non-terraced plots. However, data support the hypothesis that terraced plots acted as a buffer against the 2015 Ethiopian drought, while contour bunds did not. This study provides evidence that terraces have the potential to help farmer deal with current climate risks. These results can inform the design of climate change adaptation policies and improve targeting of soil water management practices in Ethiopia.

## Highlights

• Following a strong El-Niño, some regions of Ethiopia experienced major droughts during the 2015/16 agricultural season.
• This study investigates the effect of terraces and contour bunds on yields during the 2015 drought.
• At the national level, terraced plots have slightly lower yields than non-terraced plots.
• Terraced plots acted as a buffer against the 2015 Ethiopian drought, while contour bunds did not.
• This study provides evidence that terraces have the potential to help farmer deal with current climate risks.

## 1 Introduction

Research suggests that conventional agriculture creates unsustainable erosion rates that can result in decreased agricultural potential (Montgomery, 2007). In Ethiopia, soil erosion is considered a severe constraint for land resource productivity. Since the 1980s, massive soil conservation programs have been carried out by governmental and non-governmental organizations. Following a top-down approach, soil water management practices have been promoted according to the land’s physical limitations and erosion risks. These interventions, often implemented at the water catchment level, are designed to foster community labor mobilization (Desta et al., 2005).

Climate change is an additional burden to these already prevalent environmental challenges. Expected changes in the frequency and occurrence of precipitation patterns in Ethiopia (Souverijns et al., 2016) are likely to threaten agricultural productivity (Sultan, 2012). In recent years, climate-smart agriculture has emerged as an approach whose objective is to integrate climate change into the planning and implementation of sustainable agricultural strategies (Lipper et al., 2014). Three pillars define this approach: sustainable production; adapting and building resilience to climate change; and developing opportunities to reduce greenhouse gas emissions from agriculture. Consequently, soil water management practices that sustainably increase productivity and resilience, while reducing greenhouse gas emissions certainly qualify as climate-smart practices (Zougmoré et al., 2014). Although there is considerable interest in understanding farmer’s resilience to climate change among different soil water management practices, surprisingly little empirical evidence exists. The question of whether soil water management practices can increase resilience in the context of climate change can best be assessed through crop yield response to extreme events.

This article explores two soil water management practices that have been widely adopted in Ethiopia: terraces and contour bunds. Farmers implement these practices as an adaptive strategy for agricultural land use in mountainous areas prone to erosion. They differ regarding the scale of intervention needed, the amount of labor involved and the potential benefits for yields (WOCAT, 2003). On the one hand, terraces consist of building horizontal human-made plots and are thus labor intensive. By reducing plot steepness, terraces affect soil composition, hydrology and thus plant growth. A meta-analysis found that terracing was on average 11.5 times more efficient at controlling erosion than non-terraced plots (Wei et al., 2016). The authors also observe an effect on runoff reduction, soil water recharge and nutrient enhancement. In Ethiopia, terraces have been mostly implemented in the form of level ditches – where the soil is thrown uphill to form an embankment (*Fanja yuu* terraces) – but stone bunds are also common (Kato et al., 2011). On the other hand, contour bunds are a simple agronomic measure that consists of ploughing and planting across the field slope so that it matches contour lines. The practice does not lead to changes in the slope profile and can be repeated each cropping season. Gebreegziabher et al. (2009) provide evidence of a positive effect of contour bunds on water utilization and soil conservation. As noted by the authors, contour bunds are most efficient when used in combination with other soil water management practices.

Previous studies in sub-Saharan Africa have shown mixed evidence regarding the effect of terraces and contour bunds on yields, with most, but not all studies reporting a positive effect (Gebremedhin et al., 1999; Vancampenhout et al., 2006; Adgo et al., 2013). In Tanzania, Raes et al. (2007) found that contour bunds appreciably increased the production of rain-fed lowland rice. One limitation of these studies is that they are conducted on relatively homogenous areas while also relying on small sample sizes, raising questions about inferences that can be made. Another interesting contribution is Kato et al. (2011) who found that soil water management practices perform differently in the different rainfall regimes of Ethiopia. While the authors acknowledged a risk-reducing effect of stone bunds in low-rainfall areas, soil bunds were only effective when used in combination with other technologies. At present, the relationship between soil water management and yield response to extreme events has seldom been examined and no empirical evidence exist for how yields would respond to extreme events. In 2015, some regions of Ethiopia faced one of the worst drought in decades. The zones of Tigray and Amhara were hit particularly hard (see Fig. A.1 in supplementary material). According to UN estimates, about 10.2 million farmers were in need of food assistance at the end of the 2015/16 meher season (WFPE, 2016).

The pathway through which soil water management practices may offer a buffering effect when a drought occurs is complex. Studies have demonstrated that at the global level, climate variability accounts for one third of observed cereal yields variability (Lobell and Field, 2007; Ray et al., 2015). However, due to the predominantly rainfed system of cultivation in sub-Saharan Africa, as high as 60% of cereal yield variability could be explained by climate variability (Ray et al., 2015). When rainfall events occur, part of the surface water runs-off while the rest infiltrates the soil at different levels. On mountainous plots, water infiltration in the soil is typically limited: most of surface water runs-off. Terracing decreases connectivity of overland flow, allows enhancing water infiltration and leading to an increase in soil moisture. The micro-watersheds created by terracing can increase water concentration as well as soil nutrient, which in turns provides better water conservation (Vancampenhout et al., 2006). The altered soil structure, richer in nutrients, can reduce evaporation and consequently have a shock minimizing effect when a drought occur. Thus, it is hypothesized that, compared to sites without terraces, terracing leads to a yield increase in drought-affected areas due to deeper water infiltration, soil water availability, soil nutrient supply and reduced evaporation. This effect is hypothesized to be more important on terraced plots, that rely on a structural transformation, than on plots where contour bunding was practiced. While contour bunds also decrease run-off volume, the effect on soil nutrient levels is likely more limited (Gebreegziabher et al., 2009) and thus the effect on yields may not be as pronounced as for terracing. Yields are influenced by several other factors, including biophysical factors, crop genetics, biotic and abiotic stress as well as farm management practices at different phenological stages of plant growth. Ideally, these factors should be accounted for when estimating the effect of soil water management practices on yields.

This article thus investigates the effect of terraces and contour bunds on yields during the 2015 drought in Ethiopia. Using the propensity matching method, the treatment effect of these practices is first estimated across all of Ethiopia and then stratified across agro-ecological settings. Furthermore, yield response of terraced and contour bunds plots given drought intensity is assessed.

## 2 Methods

### 2.1 Data description

**Fig. 1:**
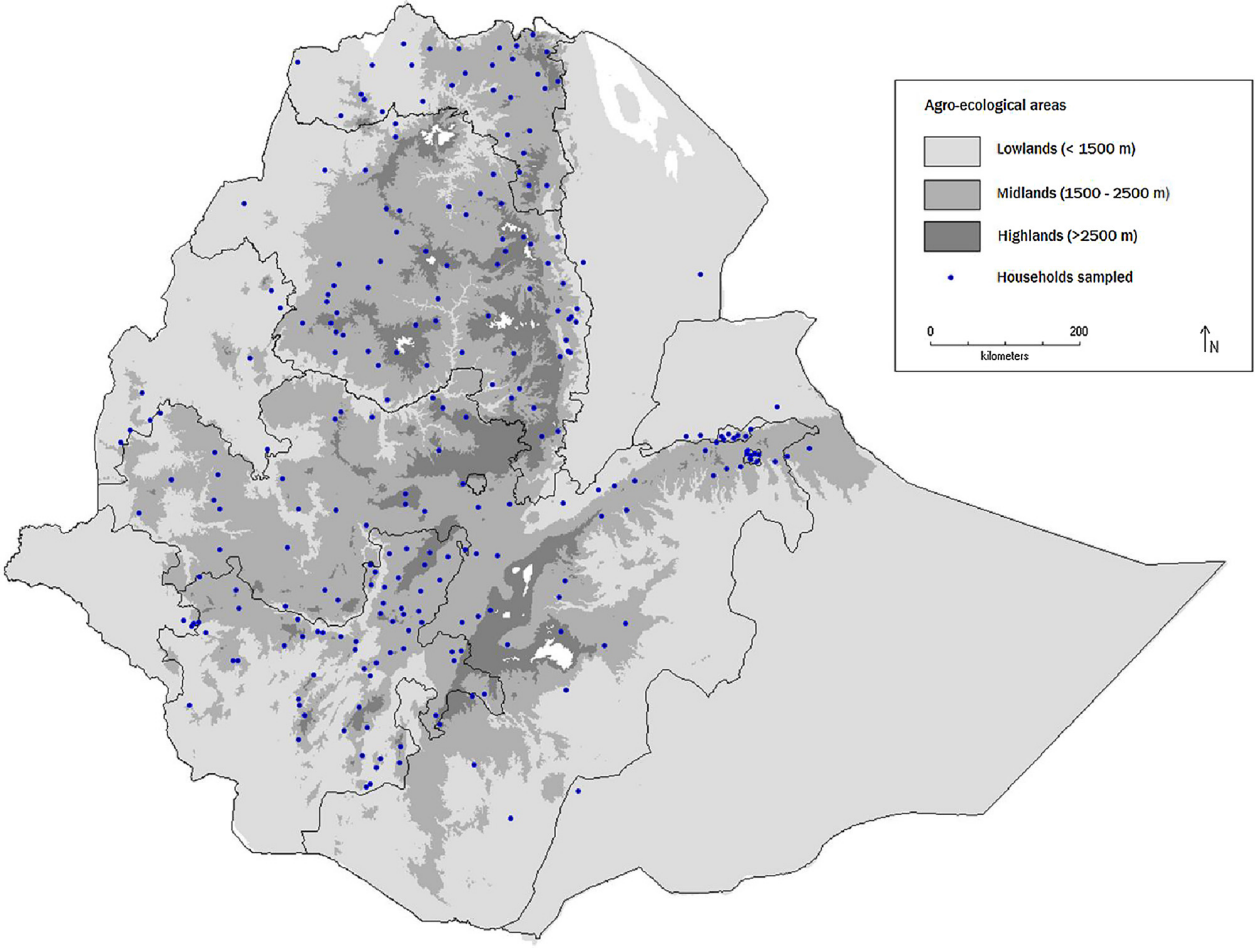
Map of Ethiopia showing agro-ecological areas and sampled households.

This study draws from the Ethiopian Socioeconomic Survey (ESS) – 2015/16. ESS is a large-scale survey with a strong focus on agriculture. It draws on a nationally and regionally representative sample of 5500 households living in rural and urban areas. The survey was implemented by the Central Statistical Agency of Ethiopia in collaboration with the World Bank Living Standards Measurement Study (LSMS) team. Households were selected for the survey through a random two-stage process. In the first stage, 433 Enumeration Areas (EAs) were selected with Probability Proportional to Size. In the second stage, 12 households were chosen randomly in each rural and small town EAs (Fig. 1). Data were collected from September 2015 to April 2016. Datasets are publicly available at http://microdata.worldbank.org/index.php/catalog/2783.

For precipitations, daily rainfall values from the Climate Prediction Center’s (CPC) unified gauge-based analysis, available at a 0.5 ° resolution for Ethiopia were extracted using the “raincpc” package (Goteti, 2014) in R. The dataset contains gauge reports from over 30,000 stations over the global land areas. After a quality control process based on historical records and alternative sources (nearby stations, satellite observations and model forecasts), gauge stations outputs are interpolated with consideration of orographic effects, as described in Xie et al. (2007). Monthly mean Temperature data were obtained from the National Oceanic & Atmospheric Administration (NOAA) GHCN CAMS global land surface temperatures dataset (Fan and van den Dool, 2008), also available at a 0.5 ° resolution for Ethiopia. Rainfall and temperature data were estimated for each field cultivated during the 2015/16 agricultural season by every household from the ESS survey, using the 0.5 ° pixel in which each field is located. The dataset A.1 file in supplementary material contains the R code for data extraction, preparation and all computations presented in the manuscript and supplementary material while dataset A.2 contains the final dataset used for analysis.

### 2.2 Propensity score matching

As farmer’ adoption of soil water management practices is not random, an estimation of the causal effect of adoption would suffer from a selection bias: adopters and non-adopters differ in observable and non-observable characteristics that may also affect the outcome of interest. Although controlled randomization is desirable, in our case such data would be difficult to obtain under non-experimental conditions and costly to generate on a large scale.

Here, a propensity score matching method is employed in an attempt to approximate the virtues of randomization (Rosenbaum and Rubin, 1983). The method is used to match treated plots (terraced plots; plots with contour bunds) with households and fields that have similar observable characteristics but were not treated (unterraced plots; plots without contour bunds). In the matching process, all covariates that are likely to affect the selection of a plot in the treatment group as well as covariates likely to influence yields were included (Heckman et al., 1997). Covariates that are thought to be affected by treatment were not included. Matching was performed using 30 covariates that include household, environment and plot level characteristics, based on a review of the existing literature (Amsalu and de Graaff, 2007; Abebe and Sewnet, 2014). In order to match plots that have similar agro-ecological settings, the EA is used as a covariate. In cases where there was no similar, but untreated plot available within the EA, the spatially closest plot with similar covariates was chosen. Table A.1. in the supplementary material presents descriptive statistics on the 30 covariates used to create propensity scores. It is clear that adopters and non-adopters differ regarding their observable characteristics. By conditioning matching on these covariates it is argued that the most obvious selection effects are mitigated. Propensity score matching was conducted using the “Matching” package (Sekhon, 2011) and was estimated using a generalized linear model with logit link. Treated plots were matched to non-treated plots with the closest propensity score on a ratio of 1:1 using the nearest neighbor method algorithm with no replacement. The potential average effect of treated plots on yields is estimated by calculating the difference between the yields of treated plots and what the yields would have been if the plot had not been treated. This is done by calculating the Average Treatment on the Treated (ATT) on matched samples. As we are interested in measuring the effect of the treatment of interest (T = 1) relative to an absence of treatment (T = 0) given covariates X, we want to estimate:

ATT = E[*Y*_1_ −* Y*_0_|X, T = 1]

Where *Y*_1_ is the yield outcome for treated plots and *Y*_0_ is the yield outcome for matched plots. The ATT is first estimated for Ethiopia and then by stratifying data into agro-ecological settings and drought intensity. Findings are robust to the use of more restrictive matching methods (see Table A.3 in supplementary material).

### 2.3 Drought exposure

Two metrics of drought exposure – percentage of dry days and percentage of dry spells – were computed for each household’s location and matched with the planting and harvesting dates provided by the household survey. To ensure sufficient plant growth, the distribution of rainfall throughout the growing season is of critical importance – a proxy that is not captured by the percentage of dry days. Thus, a common indicator of drought consists of measuring dry spells, defined here as the occurrence of seven consecutive days with less than 0.3 mm of rain. Dry spells were identified using the “seas” packages (Toews et al., 2007) in R. To characterize the severity of exposure, drought indicators were then ordered into three parts, each tercile containing a third of the plots. The following categories were used: low (>48%), moderate (48–66%) and high (>66%) percentage of dry days during the growing period and low (>13%), moderate (13–27%) and high (>27%) percentage of dry spells during growing period.

### 2.4 Outcome

The primary outcome is change in yields per hectare measured by crop-cuts. In each EAs, five crop plots were randomly selected for each crop. In cases where there were less than five fields per crop, all the plots were selected for crop cutting. On the selected plots, enumerators were asked to numerate corners, starting from the closest North-West side. The entry point to the crop-cut location was then determined using a random table based on the length of the short and long sides of the plot. Enumerators walked a random distance in the direction of the long side first, then in direction of the short side. The reached location was then used as a starting point to delimitate the 4 m * 4 m quadrant and perform the crop-cuts. Once separated from the straw, the grains were dried and weighted. The analysis is restricted to crop-cutted plots of barley, maize, teff, sorghum and wheat. These crops were selected because they represent key crops in the Ethiopian agriculture in terms of acreage, volume and food security (Central Statistical Agency, 2016). Our final dataset include 1388 households with 2611 plots.

## 3 Results

At the national level, slightly less than one third of sampled plots are terraced – this figure is similar for contour bunds. Tigray and Amhara are the two regions where terraces have seen the highest adoption rates, with 55% and 46% respectively. Contour bunds are more important in Tigray (41%) and SNNP regions (40%). No significant differences exist in terms of adoption rates between agro-ecological areas.

### 3.1 Propensity scoring

Several covariates were associated with treatments (Table A.1 in supplementary material). Households that cultivate terraced plots have a better access to extension services (85% vs 78%, p = <.0001). However, these household are generally less educated (1.4 years vs 2 years, p = .0001), have access to credit to a lesser extent (21% vs 27%, p = .01) and own less oxen (1.37 vs 1.55, p = .0001) than household that do not cultivate terraced plots. Environmental factors associated with terracing include higher altitudes (2090 m vs 2039 m, p = .01), hotter temperatures (20.3 °C vs 19.8 °C, p = .0001) and higher rainfall (481 mm vs 425 mm, p = <0.0001). Regarding plot management, terraced plots are usually smaller (1774 m^2^ vs 2099 m^2^, p = .01), less improved varieties are grown (6% vs 9%, p = .05) and manure or compost are applied more often (26% vs 21%, p = .01).

Households that have adopted contour bunds show completely different characteristics than terracing adopters. These households are more educated (2 years vs 1.7, p = .05) and own more oxen (1.62 vs 1.43, p = .001). They also have a lower access to extension services (78% vs 82%, p = .05). Contour bunds plots are also located at lower altitude (1993 m vs 2086 m, p = <.0001) while being larger (2297 m^2^ vs 1849 m^2^, p = .0001). Contour bunds plots are also more often practiced on vertisol soils (38% vs 33%, p = .01).

Analysis using a logistic regression model (N = 2611) confirmed the association of several household and environmental covariates with both treatments (Table A.2 in supplementary material). Only a few covariates related to plot management were associated with the treatments. Since plot management decisions are important factors in explaining yield variability, these covariates were kept to perform matching. Matching diagnostics, available in Fig. A.1 and A.2 in supplementary material, demonstrate that matching has been successful in balancing covariates.

### 3.2 Effect of terracing and contour bunds on yields

**Fig. 2:**
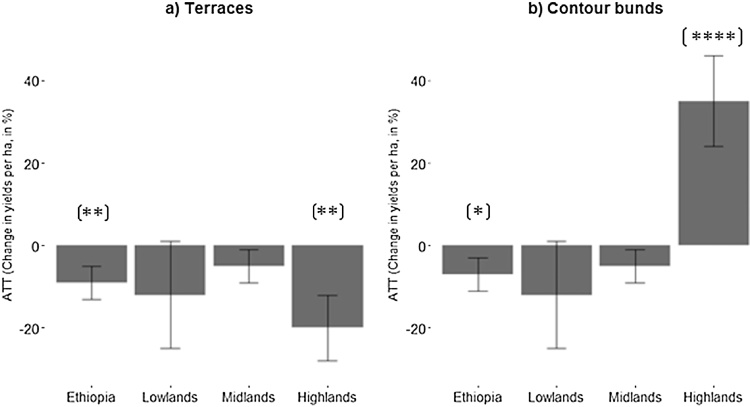
Percentages changes in yields per hectare for a) terraced plots given the agro-ecological area and (b) plots with contour bunds given the agro-ecological area. The percentage change shows the ATT estimates divided by the mean cereal yields per ha (1379 kg). Error bars represent the standard errors. *, ** and **** refer to the .1, .05 and .001 levels of statistical significance.

In Fig. 2a, ATT estimates for terraces and contour bunds plots are reported. Using the propensity score matched dataset (n = 1738), there is a slight but significant reduction in cereal yields between terraced and unterraced plots at the national level (−9.5%, p = .05). This slight yield penalty was also apparent when comparing plots with contour bunds to plots without (−7%, p = .1), as shown in Fig. 2b. However, this effect is not robust to the use of a more restrictive caliper (Table A.3 in supplementary material).

When broken down by agro-ecological areas, the difference between terraces and non-terraced plots was negative and significant in the highlands (>2500 m). Yields were also significantly higher on contour bunds plots compared to non-contour bunds plots in the highlands. Thus, at this stage of analysis, there is no evidence that terraced and contour bunds plots perform better in water-limited and drought-prone areas that are typically found in lowland areas.

### 3.3 Yields of terraced and contour bunds plots on drought-affected areas

**Fig. 3:**
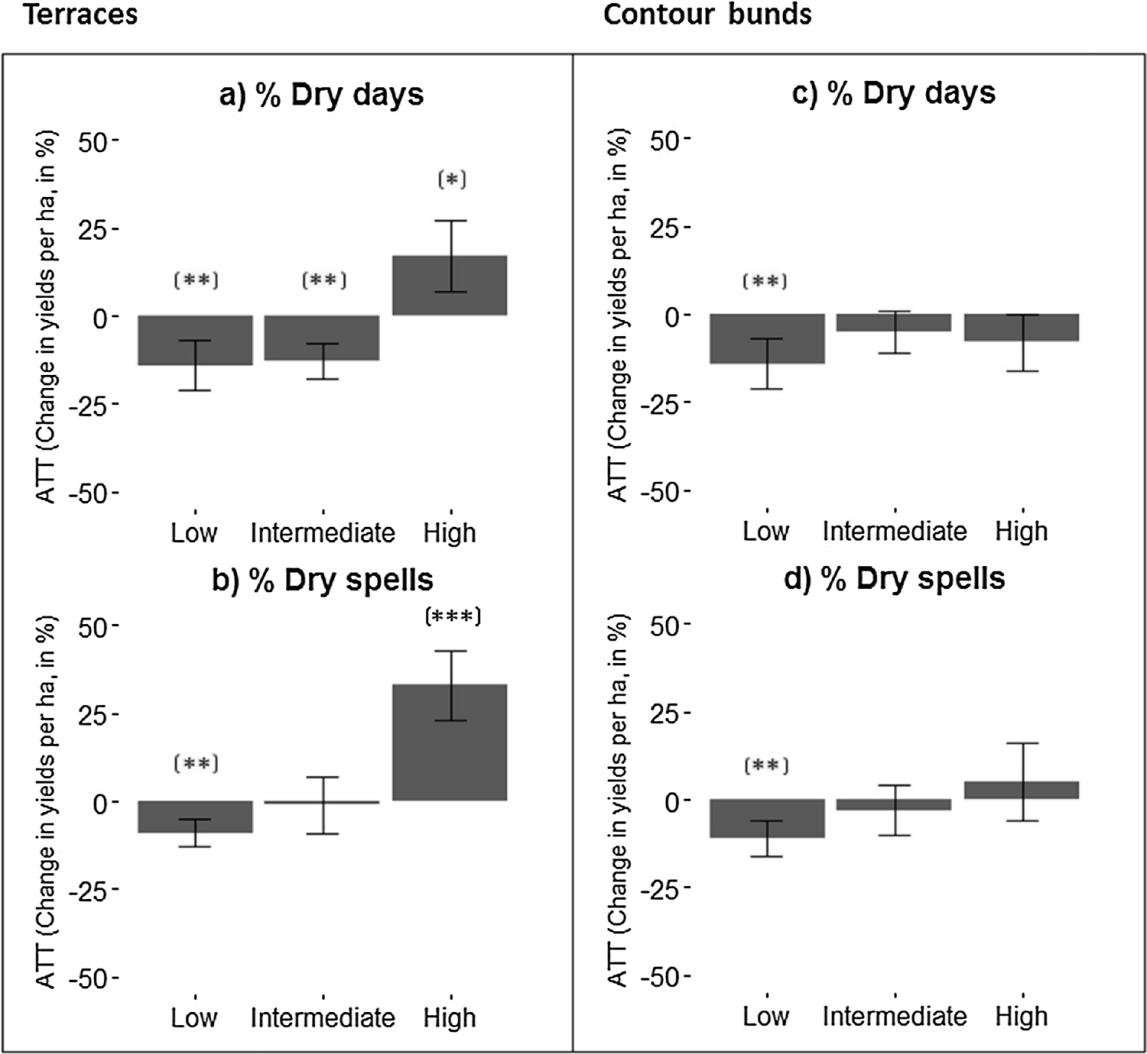
Percentages changes in yields per hectare for a) terraced plots given the % of dry days during crop growth length; (b) terraced plots the % of dry spells during crop growth length; c) plots with contour bunds given the % of dry days during crop growth length and d) plots with contour bunds given the % of dry spells during crop growth length. Estimates are based on nearest neighbor matching without caliper. The percentage change shows the ATT estimates divided by the mean cereal yields per ha (1379 kg). Error bars represent the standard errors. *, ** and **** refer to the, .05 and .001 levels of statistical significance.

Fig. 3a shows differences in yields between terraced and matched unterraced plots, revealing the treatment effect of terracing when the percentage of dry days is low (−14%, p = .05), intermediate (−13%, p = .05) and high (+17%, p = .1). When taking the consecutive number of dry days into account (dry spells), the relationship is even more pronounced. Fig. 3b indicates that yields were significantly lower on plots that experienced a low percentage of dry spells (−9%, p = .05) and significantly higher on plots with a high number of dry spells compared to unterraced plots (+33%, p = .01). As it is clear from Fig. 3c and b, no significant relationship was found between contour bunds plots and their control given drought intensity.

## 4 Discussion and conclusion

In this article, the treatment effect of terracing and contour bunds on yields was estimated using propensity score matching on a dataset representative of Ethiopia. Three key insights have emerged from this study. First, results showed that terraced plots have significantly lower yields than unterraced plots, confirming and extending previous findings (Shiferaw and Holden, 2001; Adimassu et al., 2012). This slight yield penalty (−9.5%) is consistent with the surface reduction occupied by benches or stones. Second, we found little evidence to suggest that treatment effects differ by agro-ecological areas, a finding that contradicts the results of Kato et al. (2011). This conflicting finding could be attributed to the difference in proxies used for rainfall amount. Kato et al. (2011) utilized historical rainfall data. Here, rainfall amount is matched with planting and harvesting dates of each surveyed plots. Another result, consistent with Kato et al. (2011) is that contour bunds have the potential to increase yields in highlands. However, contour bunds should be used in combination (Gebreegziabher et al., 2009; Kato et al., 2011), while terracing stands out as a potentially independent practice. When terraces are not possible, a combination of soil water management practices should thus be recommended when designing climate change adaptation plans. Third, the results support our main hypothesis that terraces, carried out primarily to control water runoff and erosion, acted as a buffer during the 2015 Ethiopian drought. Contour bunds, a less intensive soil water management practice, did not minimize drought impacts. These findings demonstrate the high relevance of terraces for climate-smart agriculture. While available evidence suggests that terracing offers benefits for sustainability (Vancampenhout et al., 2006; Raes et al., 2007; Adimassu et al., 2012; Adgo et al., 2013), this study brings further evidence that terraces can help farmers to build resilience in the face of extreme events. Indeed, trade-offs exist between the risk mitigation benefits of terracing with the yield reduction in years with adequate rainfall. In drought-prone areas, the practice should be promoted as a way to increase farmer’s adaptive capacity.

This study is not without limitations. An obvious concern is that propensity matching is able to control observable characteristics only; one cannot be certain regarding unobserved variables. Since community mobilization and labor investment are important factors for terracing, adoption is largely driven by program implementations and is therefore mostly exogenous. This minimizes the concern that adoption could suffer from endogeneity bias related to unobserved characteristics. This limitation notwithstanding, the study has a number of strengths. First, it relies on a representative dataset of Ethiopia and uses a robust measurement methods – crop-cuts – as the outcome metric. This measure is arguably more accurate than farmers’ yield estimates – a widely used proxy in the literature. In addition, rainfall and drought occurrences were precisely matched with plot-level crop growing period. Further, several efforts were made in order to create a credible counterfactual. The number of covariates as well as matching diagnostics led us to conclude that a proper counterfactual was established. Sensitivity analysis showed that the estimated effect held regardless of matching within or outside the common support region.

Overall, this study contributes to the literature on climate-smart practices by providing our best estimation at this point regarding the contribution of soil water management practices to resilience against extreme events. Given that adaptation benefits from terracing could be larger than previously thought, these results can help designing scenarios about climate change impacts. Bearing in mind that most terraces can be found in the Tigray and Amhara regions, the extension of terraces could also be considered as part of the country’s strategy to mitigate effects of extreme events in other regions beyond where they are currently found.

## Appendix A Supplementary data

Fig and Tables - R code - Dataset:
