# Supplementary material for: Soil water management practices (terraces) helped to mitigate the 2015 drought in Ethiopia
Source: Agric Water Manag. 2018 May 31;204:11–6. doi: 10.1016/j.agwat.2018.02.025 (PMC5985715; doi:10.1016/j.agwat.2018.02.025)

Table A.1. Covariates definitions and descriptive statistics by treatment status.

Variable	Description	Terraces			Contour bunds		
		Adopter mean	Non-adopter mean	*t*-test	Adopter mean	Non-adopter mean	*t*-test
**Household**							
Age of household head	Age of HH head in years	49.7 (13.7)	48.1 (14)	0.0046	48.2 (13.9)	48.8 (13.9)	0.2493
Head education	HH head number of years of scholing	1.41 (2.6)	2.04 (3.1)	0.0000	2.04 (3)	1.74 (2.9)	0.0187
Household size	Household size	6.9 (2.3)	6.9 (2.4)	0.3946	6.86 (2.4)	6.97 (2.4)	0.2983
Oxen ownership	Number of oxen owned by household	1.37 (1)	1.55 (1.29)	0.0001	1.62 (0.43)	1.43 (0.43)	0.0002
Access to extension services	HH head has access to agricultural advisory services	0.85 (0.36)	0.78 (0.41)	0.0000	0.78 (0.41)	0.82 (0.39)	0.0319
Access to credit	HH head has access to credit	0.21 (0.41)	0.27 (0.44)	0.0016	0.25 (0.43)	0.25 (0.43)	0.9743
Community participation	HH head participates in watershed activities within the EA	0.83 (0.37)	0.78 (0.41)	0.0005	0.79 (0.41)	0.81 (0.40)	0.2097
**Environment**							
Enumeration Area	Primary sampling unit ID	--	--	--	--	--	--
Altitude	Household dwelling elevation, in meters	2090 (448)	2039 (442)	0.0068	1993 (446)	2086 (441)	0.0000
Temperature	Mean temperature during crop growth, in C°	20.3 (3.6)	19.8 (3.5)	0.0001	20.1 (3.4)	19.9 (3.6)	0.0567
Rainfall amount	Total rainfall amount during crop growth, in mm	481 (238)	425 (253)	0.0000	445 (278)	440 (235)	0.6468
**Plot management**							
Plot size	Size of plot in square meters, measured by GPS	1774 (2913)	2099 (2554)	0.0062	2297 (2995)	1849 (2488)	0.0001
Plot is flat	Dummy for plot is flat	0.51 (0.50)	0.58 (0.49)	0.0022	0.58 (0.49)	0.54 (0.50)	0.0380
Plot has moderate slope	Dummy for plot has moderate slope	0.38 (0.49)	0.33 (0.30)	0.0070	0.31 (0.46)	0.36 (0.48)	0.0051
Plot has steep slope	Dummy for has steep slope	0.10 (0.31)	0.10 (0.30)	0.4533	0.11 (0.31)	0.10 (0.30)	0.3557
Soil type is cambisol	Dummy for soil is cambisol	0.03 (0.18)	0.02 (0.15)	0.2952	0.03 (0.16)	0.03 (0.16)	0.8578
Soil type is leptosol	Dummy for soil is leptosol	0.13 (0.33)	0.09 (0.29)	0.0088	0.10 (0.30)	0.11 (0.31)	0.4199
Soil type is luvisol	Dummy for soil is luvisol	0.36 (0.48)	0.32 (0.47)	0.0302	0.31 (0.46)	0.34 (0.47)	0.1267
Soil type is vertisol	Dummy for soil is vertisol	0.26 (0.44)	0.38 (0.49)	0.0000	0.38 (0.49)	0.33 (0.47)	0.0065
Soil type is mixed	Dummy for soil is mixed	0.17 (0.37)	0.15 (0.35)	0.1894	0.15 (0.36)	0.15 (0.36)	0.7161
Other Soil type	Dummy for soil is other	0.04 (0.21)	0.03 (0.37)	0.2727	0.03 (0.17)	0.04 (0.20)	0.1759
Crop type is barley	Dummy for crop is barley	0.16 (0.37)	0.16 (0.37)	0.9003	0.16 (0.37)	0.16 (0.37)	0.8105
Crop type is maize	Dummy for crop is maize	0.19 (0.39)	0.26 (0.44)	0.0000	0.25 (0.43)	0.23 (0.42)	0.4987
Crop type is sorghum	Dummy for crop is sorghum	0.26 (0.44)	0.17 (0.37)	0.0000	0.18 (0.38)	0.20 (0.40)	0.1873
Crop type is teff	Dummy for crop is teff	0.22 (0.41)	0.24 (0.42)	0.2293	0.25 (0.43)	0.22 (0.41)	0.1361
Crop type is wheat	Dummy for crop is wheat	0.18 (0.38)	0.18 (0.38)	0.9049	0.17 (0.37)	0.18 (0.38)	0.3923
Irrigation	Plot is irrigated by pump or river diversion	0.03 (0.17)	0.01 (0.08)	0.0003	0.01 (0.10)	0.01 (0.12)	0.2570
Improved variety	Crop planted on plot is an improved variety	0.06 (0.24)	0.09 (0.28)	0.0151	0.08 (0.27)	0.08 (0.27)	0.7466
Inorganic fertilizer	Quantity of DAP /UREA applied on plot, in kg per ha	364 (3519)	283 (2833)	0.6084	159 (852)	383 (3699)	0.0160
Organic fertilizer	Manure or compost applied on plot	0.26 (0.44)	0.21 (0.41)	0.0139	0.21 (0.41)	0.23 (0.42)	0.5700

Note: numbers in parenthese are standard errors.

Table A.2. Estimates of the logit models.

Covariate	Terraces		Contour bunds	
	Estimate	z value	Estimate	z value
Age of household head	0 (0)	1.33	0 (0)	-0.49
Head education	-0.06 (0.02)	-3.51****	0.03 (0.02)	2.29**
Household size	0.01 (0.02)	0.68	-0.06 (0.02)	-3.17***
Oxen ownership	-0.15 (0.04)	-3.4****	0.15 (0.04)	3.83****
Access to extension services	0.56 (0.13)	4.29****	-0.23 (0.11)	-2.03**
Access to credit	-0.28 (0.11)	-2.59***	-0.01 (0.1)	-0.11
Community participation	0.5 (0.13)	3.84***	-0.14 (0.11)	-1.26
Altitude	0 (0)	4.95****	0 (0)	-5.13****
Temperature	0.12 (0.02)	6.69****	-0.01 (0.02)	-0.58
Rainfall amount	0 (0)	2.85***	0 (0)	2.94***
Plot size	0 (0)	-2.1**	0 (0)	2.78***
Plot has moderate slope (Ref = flat)	0.36 (0.1)	3.59****	-0.18 (0.09)	-1.85*
Plot has steep slope (Ref = flat)	0.18 (0.16)	1.16	0.07 (0.15)	0.51
Soil type is leptosol (Ref = cambisol)	0.14 (0.31)	0.47	-0.02 (0.29)	-0.08
Soil type is luvisol (Ref = cambisol)	0.05 (0.28)	0.16	0 (0.27)	0
Soil type is vertisol (Ref = cambisol)	-0.38 (0.29)	-1.3	0.26 (0.27)	0.97
Soil type is mixed (Ref = cambisol)	-0.06 (-0.06)	0.29	0.04 (0.04)	0.28
Other Soil type (Ref = cambisol)	0.29 (0.36)	0.81	-0.28 (0.35)	-0.81
Crop type is maize (Ref = barley)	-0.23 (0.17)	-1.35	-0.27 (0.16)	-1.74*
Crop type is sorghum (Ref = barley)	0.75 (0.17)	4.42****	-0.54 (0.16)	-3.32****
Crop type is teff (Ref = barley)	0.21 (0.16)	1.38	-0.18 (0.15)	-1.21
Crop type is wheat (Ref = barley)	0.16 (0.16)	1.02	-0.15 (0.15)	-0.98
Irrigation	1.88 (0.4)	4.74****	-0.71 (0.41)	-1.73*
Improved variety	0.12 (0.2)	0.62	0.01 (0.17)	0.03
Inorganic fertilizer	0 (0)	0.7	0 (0)	-1.36
Organic fertilizer	0.07 (0.12)	0.61	0.05 (0.11)	0.44
EA-fixed effects	0 (0)	-8.04****	0 (0)	-0.76
Intercept	-5.18 (0.73)	-7.13****	1.39 (0.68)	2.05**
N	2611		2611	
Count R2	72%		67%	

Note: numbers in parenthese are standard errors

*, **, ***,**** Statistically significant at the 0.1, 0.05, 0.01 and 0.001 level respectively.

Table A.3. Results of the ATTs using calipers that are more restrictive.

	Change in yields per ha		Change in yields per ha with caliper .05		Change in yields per ha with caliper .01	
	ATT	p-value	ATT	p-value	ATT	p-value
**Terraces**						
Ethiopia	-121 (52)	0.0206	-142 (58)	0.0142	-165 (58)	0.0044
**Agro-ecological area**						
Lowlands	-162 (174)	0.3496	-104 (203)	0.6070	120 (246)	0.6242
Midlands	-74 (58)	0.1988	-34 (63)	0.5957	-114 (68)	0.0953
Highlands	-280 (113)	0.0128	-335 (146)	0.0218	-524 (167)	0.0017
**% dry days**						
Low	-187 (91)	0.0397	-124 (95)	0.1937	-138 (107)	0.1966
Moderate	-177 (72)	0.0142	-230 (80)	0.0040	-177 (72)	0.0143
High	228 (136)	0.0943	367 (177)	0.0389	176 (205)	0.3917
**% dry spells**						
Low	-119 (60)	0.0461	-134 (68)	0.0508	-168 (70)	0.0184
Moderate	-8 (110)	0.9417	4 (124)	0.9749	0 (140)	0.9972
High	456 (136)	0.0031	630 (311)	0.0427	695 (343)	0.0424
**Contour bunds**						
Ethiopia	-99 (53)	0.0642	-49 (54)	0.3685	-51 (56)	0.3629
**Agro-ecological area**						
Lowlands	-162 (174)	0.3496	-104 (203)	0.6070	120 (246)	0.6242
Midlands	-74 (58)	0.1988	-34 (63)	0.5957	-114 (68)	0.0953
Highlands	480 (145)	0.0008	378 (139)	0.0066	317 (175)	0.0692
**% dry days**						
Low	-187 (91)	0.0397	-124 (95)	0.1937	-138 (107)	0.1966
Moderate	-64 (76)	0.3994	-34 (82)	0.6762	-106 (89)	0.2293
High	-107 (113)	0.3404	60 (139)	0.6650	-19 (161)	0.9046
**% dry spells**						
Low	-152 (70)	0.0309	-155 (74)	0.0363	-110 (74)	0.1402
Moderate	-44 (96)	0.6425	-52 (102)	0.5789	-42 (114)	0.7116
High	63 (146)	0.6664	58 (200)	0.7723	399 (278)	0.1506

Figure A.1. 1-month Standardized Precipitation Evapotranspiration Index (SPEI) for Amhara, Oromiya, SNNP and Tigray regions, ending in March 2016. The 2015/16 meher season is contained within the red bars.

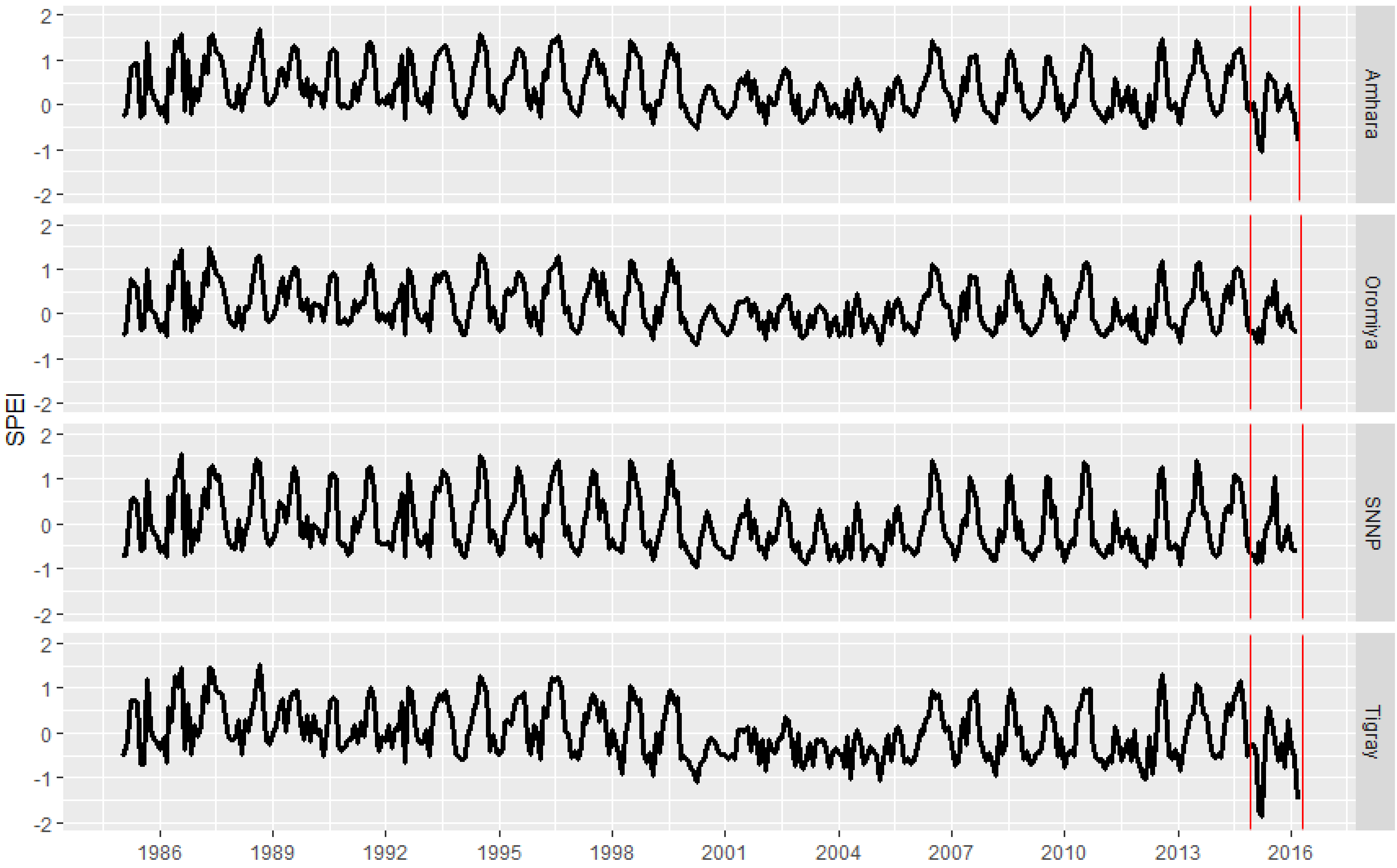

Figure A.2.a Plots of standardized difference of means of 30 covariates before and after matching given the agro-ecological area.

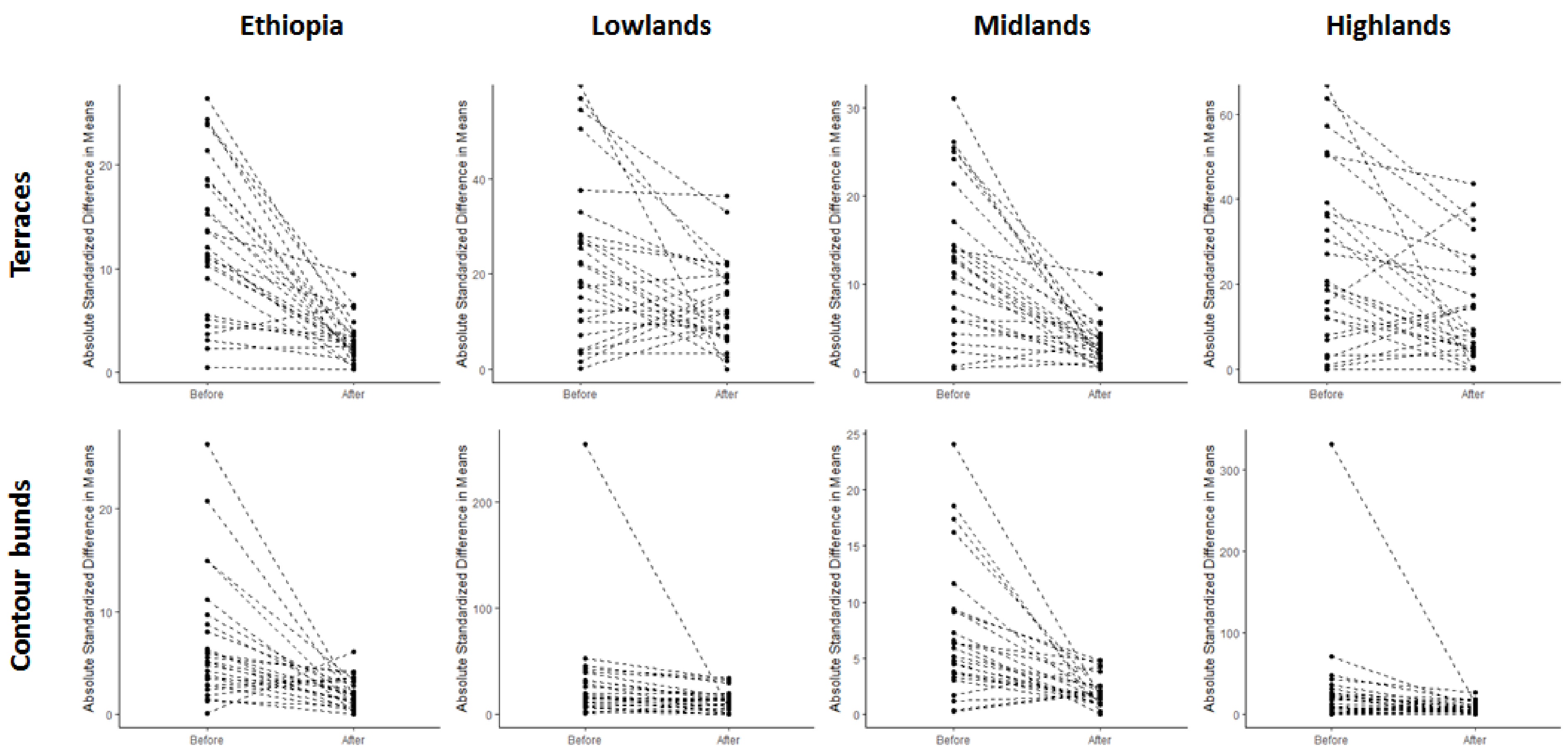

Figure A.2.b Plots of standardized difference of means of 30 covariates before and after matching given the % of dry days and the % of dry spells.

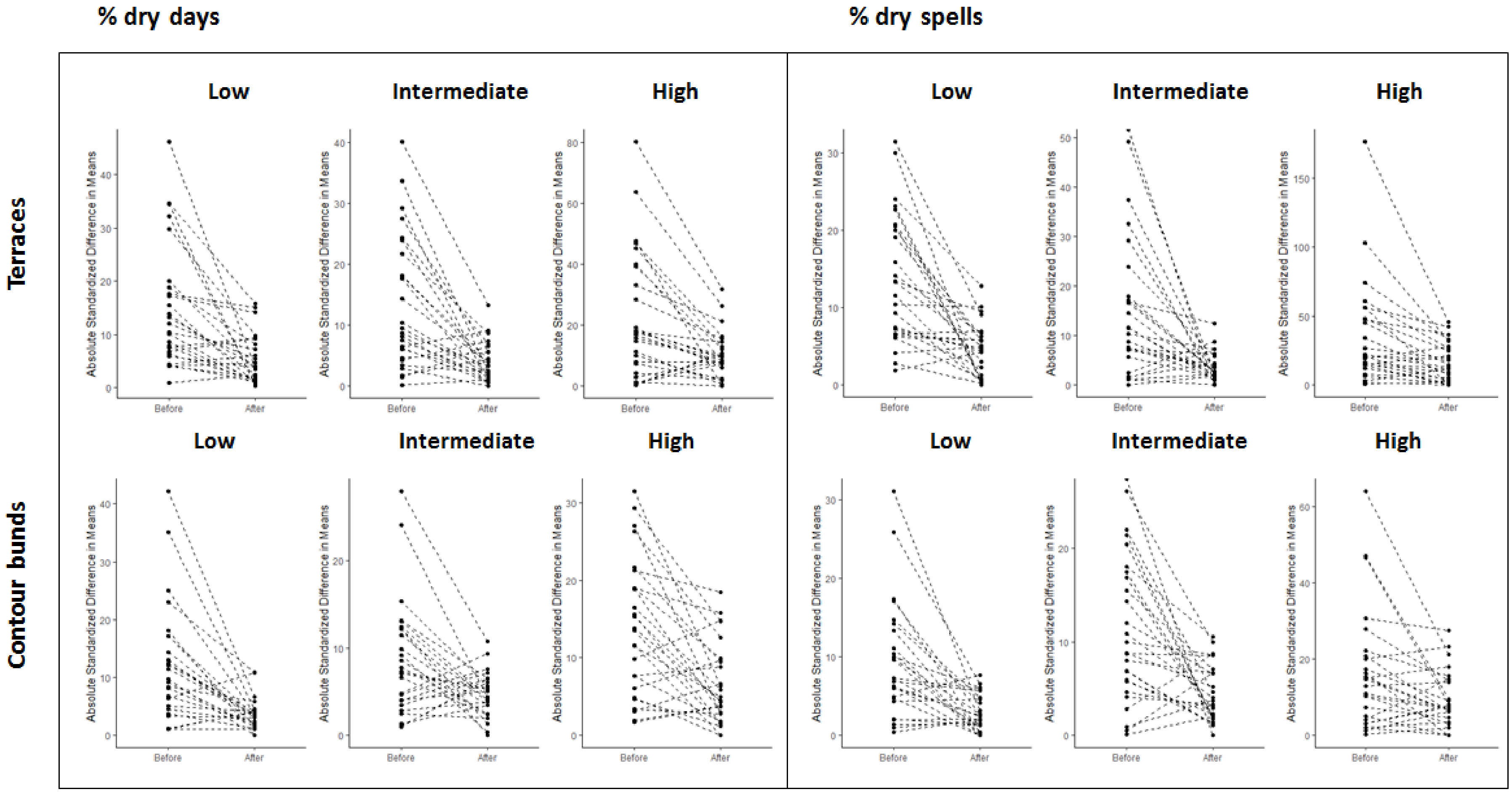

Figure A.3.a Overlay of kernel density distribution of propensity scores before and after propensity score matching given the agro-ecological area. Treated plots are shown in plain lines while control plots are represented by dashed lines.

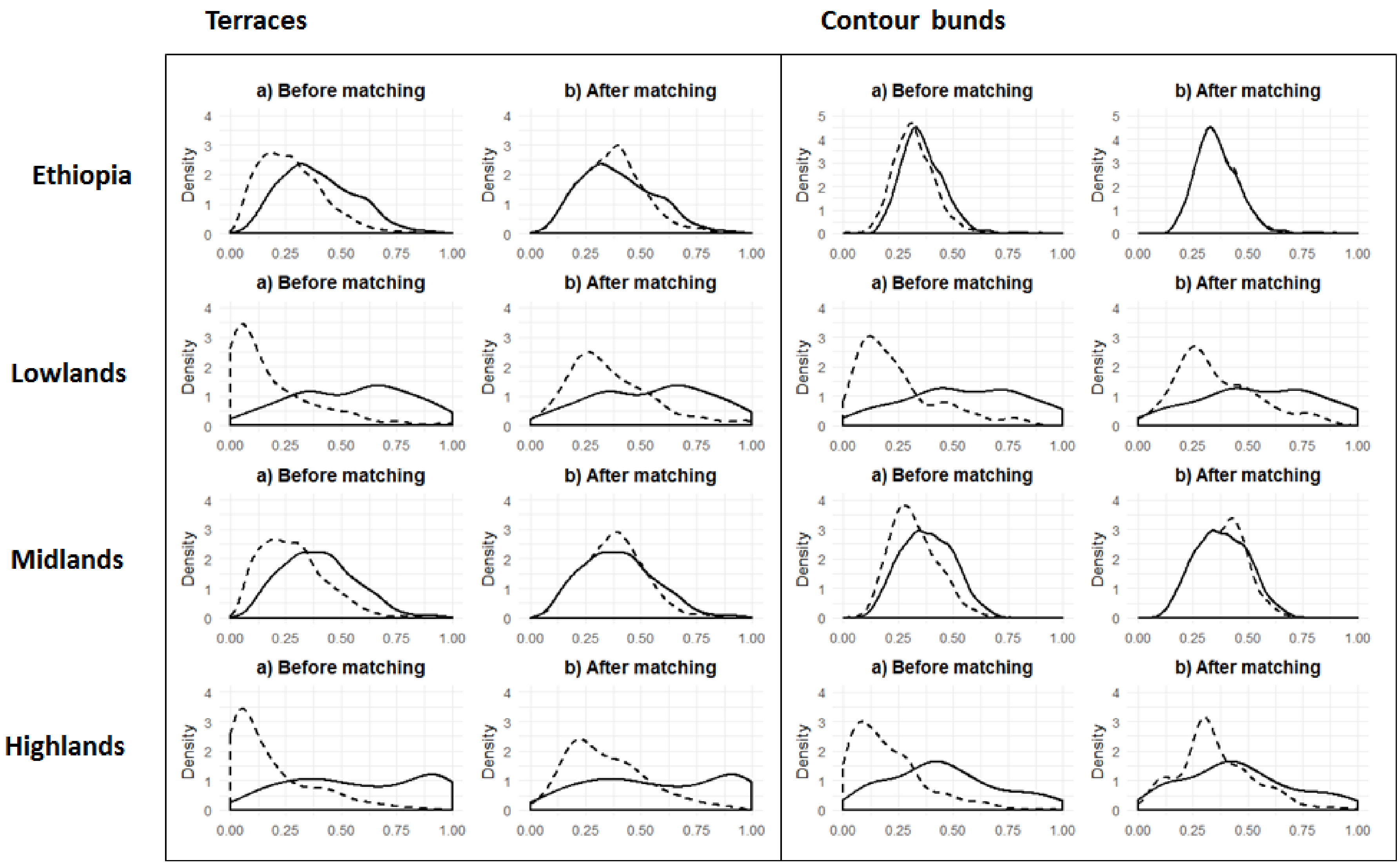

Figure A.3.b Overlay of kernel density distribution before and after propensity score matching given the % of dry days. Treated plots are shown in plain lines while control plots are represented by dashed lines.

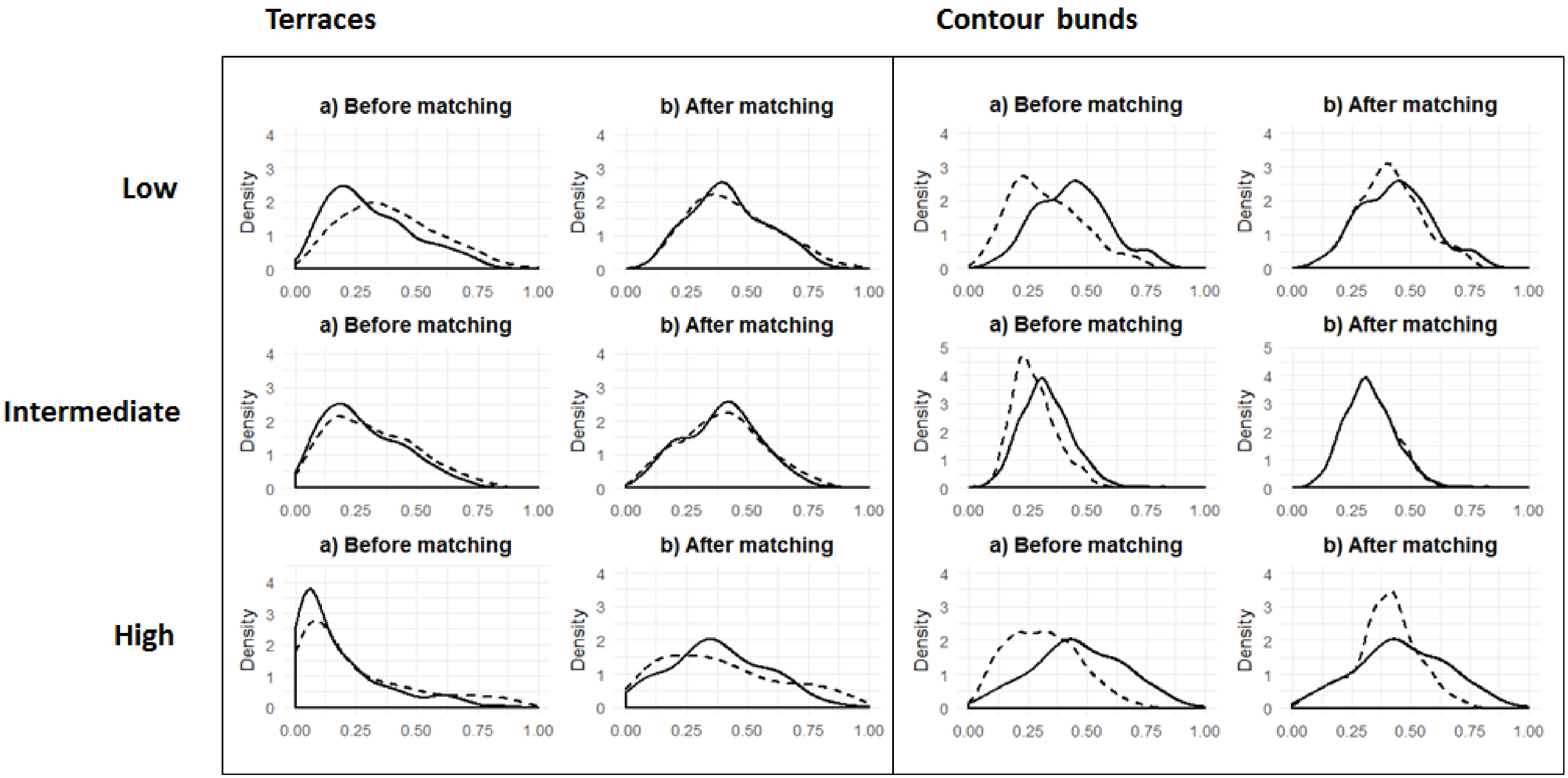

Figure A.3.c Overlay of kernel density distribution before and after propensity score matching given and the % of dry spells. Treated plots are shown in plain lines while control plots are represented by dashed lines.

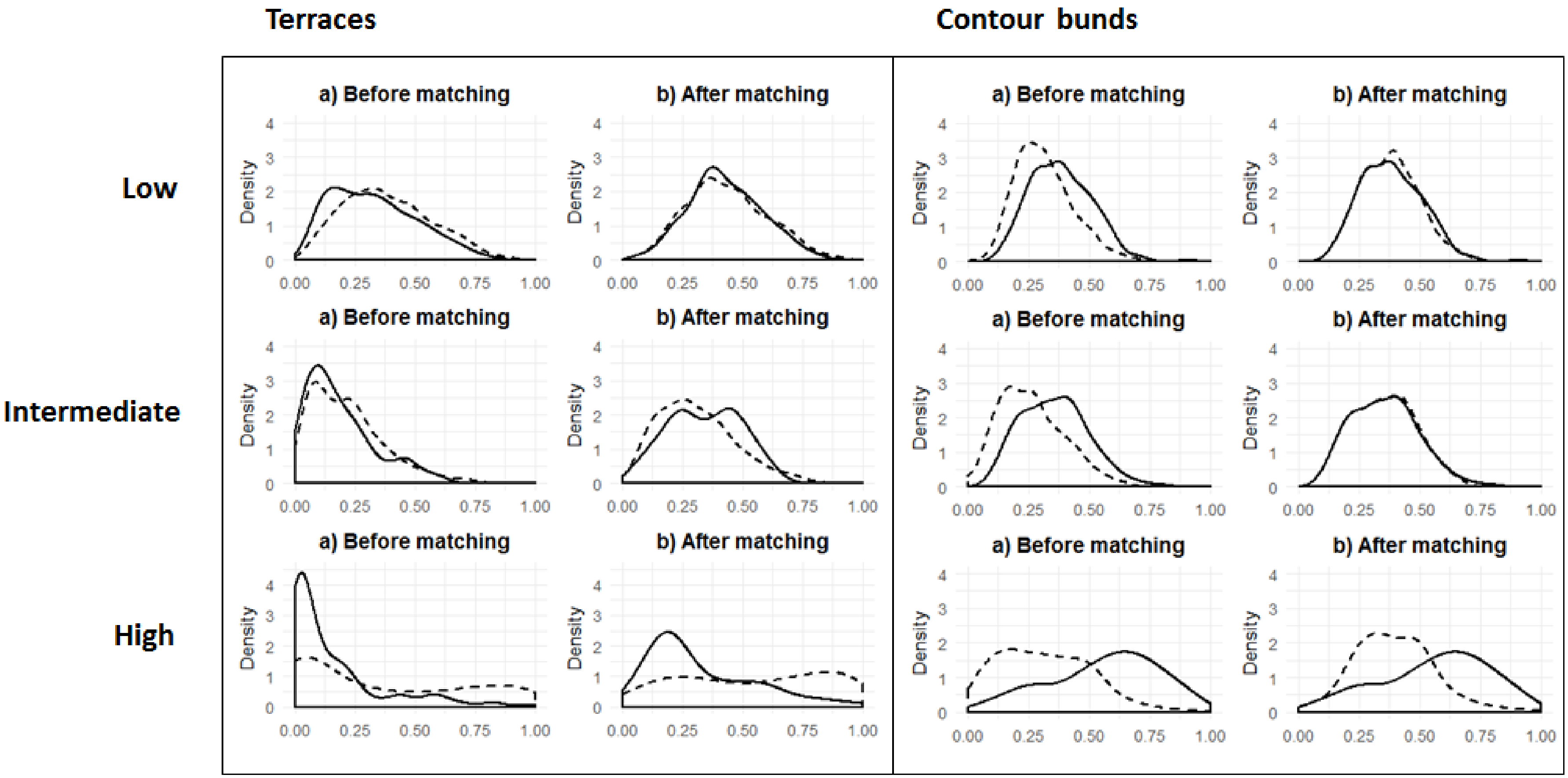

Supplement: Supplementary file 1 [file mmc1.pdf]
